# Integrating UAV multispectral imaging and proximal sensing for high-precision cereal crop monitoring

**DOI:** 10.1371/journal.pone.0322712

**Published:** 2025-05-22

**Authors:** Željana Grbović, Bojana Ivošević, Maša Budjen, Rana Waqar, Nina Pajević, Natasa Ljubičić, Vesna Kandić, Miloš Pajić, Marko Panić

**Affiliations:** 1 BioSense Institute, University of Novi Sad, Novi Sad, Serbia; 2 Breeding Department, Maize Research Institute Zemun Polje, Belgrade, Serbia; 3 Faculty of Agriculture, University of Belgrade, Belgrade, Serbia; Shandong Agricultural University, CHINA

## Abstract

Multispectral optical data significantly enhances cereal crop monitoring by enabling precise tracking of growth stages, early detection of germination issues, and assessment of plant health. This study evaluates the potential of integrating UAV multispectral sensor with the handheld Plant-O-Meter device for high-precision crop monitoring. The aim was to determine the optimal UAV imaging timing that aligns with proximal sensor measurements to improve growth stage assessments. Experiments were conducted on 41 cereal genotypes, including ancient and modern varieties, under two nitrogen top-dress dosages across 130 plots. The top ten performing genotypes were analyzed to identify resilient varieties adaptable to climate change and evolving field conditions. Our results demonstrate that vegetation indices during booting and spike emergence stages consistently predict yield potential, offering a robust framework for early-stage yield estimation. Additionally, we provide a comparative analysis of UAV and handheld sensor data, highlighting their respective strengths and limitations. Three vegetation indices, GRDVI, NDVI and SAVI demonstrated a very strong average positive correlation: 0.957, 0.954 and 0.944 across the selected genotypes from different performance levels. The combined dataset supports improved fertilization strategies, optimized seeding cycles, and identification of genotypes with stable agronomic traits. This study underscores the synergistic potential of aerial and proximal sensing technologies for next-generation cereal crop management and precision agriculture.

## Introduction

### Cereal crops and genetic diversity

Cereals are among the most widely cultivated field crops globally, providing a major source of human nutrition. They contribute over two-thirds of the world’s food supply and occupy more than 56% of cultivable land [1,2]. While modern wheat varieties dominate global production, ancient grains such as einkorn *Triticum monococcum*, emmer *Triticum dicoccum*, and spelt *Triticum spelta* continue to be cultivated for their unique nutritional properties and resilience to environmental stressors [3,4]. Similarly, old varieties of barley *Hordeum vulgare* and rye *Secale cereale* offer valuable traits that contribute to biodiversity and stress resistance. Recent comparative studies between ancient and modern wheat varieties indicate that older cultivars possess superior adaptability to adverse conditions, including abiotic and biotic stress resistance such as poor soil quality and suboptimal climatic conditions [5,6]. Modern wheat breeding programs benefit significantly from the genetic diversity of ancestral cultivars, incorporating traits that enhance yield, resilience, and environmental adaptability. Accurate phenotyping is essential to identify and select the most promising traits for integration into modern selection programs.

### Remote sensing for high-throughput phenotyping

Crop phenotyping plays a critical role in understanding plant growth, physiology, and yield potential. Traditional phenotyping methods, however, are often labor-intensive, time-consuming, and expensive [7].

#### Technology advances and validation

Remote sensing technologies, particularly multispectral imaging, provide a high-throughput, cost-effective alternative, enabling non-destructive and scalable plant trait assessment throughout the growing season. Unmanned Aerial Vehicles (UAVs) equipped with multispectral sensors provide high-resolution imaging of research plots, capturing vegetation indices that reflect plant health, stress levels, and yield potential. The spectral signatures detected at different wavelengths (e.g., NIR, RedEdge) offer insights into plant canopy structure, nutrient status, and disease resistance [8]. Numerous studies have demonstrated the effectiveness of UAV-based phenotyping for cereals, either as a standalone method [9,10] or in combination with ground-based sensors [11–16]. As it is demonstrated in recent work [17], the role of UAV-based multispectral imagery for field-based high-throughput phenotyping has proven essential for monitoring crop growth stages throughout the growing seasons. Recent research has shown that UAV-derived Normalized Difference Vegetation Index (NDVI) measurements closely correlate with handheld sensor data, such as the GreenSeeker, for estimating biomass and grain yield variations. It has been shown that UAV multispectral and handheld sensors have been successfully used to extract phenotypic traits at the plot scale, demonstrating high consistency across different wheat genotypes and management practices [11,16]. Additionally, [18] shows that UAV-based NDVI with a 10 cm resolution is more effective than Landsat data for predicting wheat yield and grain protein content, emphasizing the importance of spatial scale in capturing field variability.

#### Applications and integration into breeding and crop models

The application of modern remote sensing technologies and machine learning techniques in breeding programs and crop modeling has enabled more precise predictions of crop performance, optimizing the selection of superior genotypes and enhancing overall crop management. The study [19] integrates UAV imagery into the WOFOST model, improving wheat growth and yield predictions by enhancing the accuracy of leaf area index (LAI) simulation, demonstrating the potential of UAV data for optimizing crop models. Similar to our study in [20], UAVs and VIs, such as NDVI, were used for high-throughput phenotyping of wheat seedling emergence. Our use of VIs to monitor crop growth stages is aligned with [21], which applied UAVs for monitoring wheat scab with multispectral data. Additionally, the integration of machine learning for crop management, as seen in [22], highlights the potential for real-time N status diagnosis, a concept we further refine at the plant level by using proximal sensors on the field. [23] demonstrated the use of machine learning for predicting yield and protein content, similar to our focus on optimizing resource usage with high-resolution data. Finally, [24] exemplifies the growing capabilities of UAVs in yield prediction, which is extended in our study to more precise monitoring of individual plant growth stages for improved management. These advancements in high-throughput phenotyping (HTP) are revolutionizing precision agriculture and breeding research by enabling continuous, objective, and large-scale crop assessments [13]

### Fertilization and reseeding in crop performance

Beyond phenotyping, agronomic practices such as fertilization and reseeding play a crucial role in crop performance. Fertilization ensures that crops receive essential nutrients at the right stages of development, particularly nitrogen (N), phosphorus (P), and potassium (K), which are critical for photosynthesis, root development, and grain formation [25]. The timing of fertilizer application significantly impacts plant health, as early applications may lead to nutrient leaching, while late applications may cause deficiencies during critical growth stages [26,27]. Similarly, reseeding—the process of replacing weak or damaged plants—affects crop establishment and yield potential [28]. Proper timing of reseeding prevents poor germination, reduces competition from weeds, and minimizes disease susceptibility [29,30]. Ensuring that both fertilization and reseeding are performed at optimal times can enhance yield stability, improve resource efficiency, and mitigate environmental impacts.

### Research gaps and study objectives

While UAV-based multispectral imaging has been widely applied in precision agriculture, few studies have systematically compared UAV data with ground-based handheld sensors to assess their complementary roles in crop monitoring. Additionally, the combined impact of fertilization strategies and reseeding practices on genotype performance remains underexplored particularly in relation to genotype-specific responses. In this study, we introduce a dual-sensor approach, combining UAV multispectral imagery and a handheld Plant-O-Meter device, to monitor cereal crop growth stages and assess genotype-specific responses. This approach integrates high-resolution aerial imaging with detailed ground-level measurements, providing a multi-scale assessment of crop health and performance. Based on these gaps, the study aims to address the following objectives:

- Evaluate the impact of fertilization and reseeding on crop yield and performance;
- Use UAV-derived multispectral data to identify high- and low-performing cereal genotypes across various growth stages;
- Compare the performance of UAV-based multispectral sensors with handheld Plant-O-Meter measurements.

By leveraging these advanced remote sensing techniques, this study aims to provide cost-effective, scalable, and time-efficient solutions for sustainable crop management and precision breeding.

## Materials and methods

### Field trial

**Fig 1:**
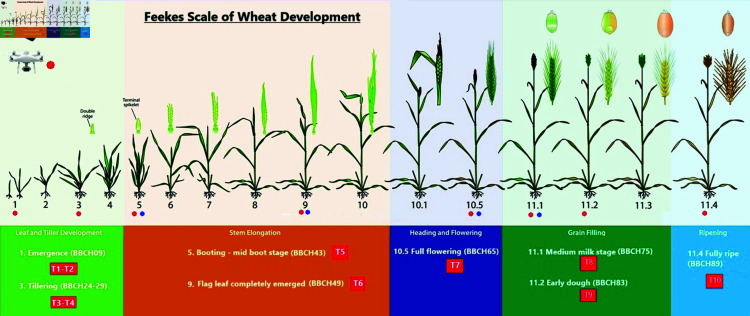
Stages of development according to the Feekes Scale and key points of the development process captured during the experiment. Red dots indicate UAV imaging events, while blue dots represent Plant-O-Meter data acquisition. The first four measurements (T1–T4) were conducted during the Leaf and Tiller phase (Emergence and Tillering stages). Measurements T5 and T6 took place during the Stem Elongation phase (Booting and Flag Leaf Emergence stages), followed by T7 during the Heading and Flowering phase (Full Flowering stage). Measurements T8 and T9 were performed during the Grain Filling phase (Medium Milk stage), and the final measurement (T10) was conducted during the Ripening phase (Fully Ripe stage).

**Fig 2:**
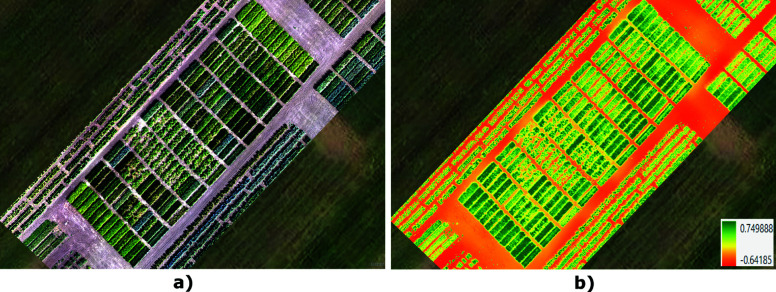
(a) RGB orthomosaic image of the experimental field; (b) NDVI map calculated across all plots.

The study was conducted in the experimental fields of the Maize Research Institute Zemun Polje (44°52’ N, 20°20’ E), Serbia, during the 2021/2022 growing season. Throughout the season, plant development was monitored using multispectral and RGB cameras mounted on a UAV, as well as a handheld proximal multispectral sensor, the Plant-O-Meter. Fig 1) provides an overview of device measurements and corresponding phenological stages, while S1 Table presents the measurement dates for both devices. Measurements with the Plant-O-Meter were conducted at only four growth stages: The booting stage (BBCH43), the stage where the flag leaf completely emerges (BBCH49), the full flowering stage (BBCH65), and medium milk stage (BBCH75) according to the BBCH Growth Stage Scale. Due to the diverse range of Poaceae species included in the experiment, some genotypes may have been at slightly different growth stages when spectral reflectance was recorded. The experiment included 43 genotypes from ten Poaceae species: barley (*Hordeum vulgare*), durum wheat (*Triticum durum*), einkorn (*Triticum monococcum*), emmer wheat (*Triticum dicoccon*), rye (*Secale cereale*), shot wheat (*Triticum sphaerococcum*), spelt (*Triticum spelta*), triticale (*Triticale*), common wheat (*Triticum aestivum*), and compact wheat (*Triticum compactum*). Many of these are ancient species, rarely cultivated commercially but valuable for plant breeding programs. A complete list of genotypes is provided in S2 Table. The field trial was conducted on a low-carbonated Chernozem soil type. Sowing took place on November 10, 2021. Each genotype was sown in four replicates, with two plots per block receiving different nitrogen treatments. A completely randomized block design (RBCD) was used to minimize soil-related variability. Each experimental plot consisted of eight rows of wheat, spaced 12.5 cm apart, with a row length of 5.4 m (Fig 2). A plant density of $600\;{seeds\;per\;m}^{2}$ was maintained. At full maturity, grain yield (t/ha) was estimated for each genotype. Additionally, individual plants were harvested to evaluate yield-related traits.

### Devices

For spectral reflectance measurements, a DJI P4 Multispectral UAV (China, Shenzhen) and an active, handheld, multispectral proximal sensor, the Plant-O-Meter (BioSense Institute, Serbia), were used.

#### Unmanned aerial vehicle

**Fig 3:**
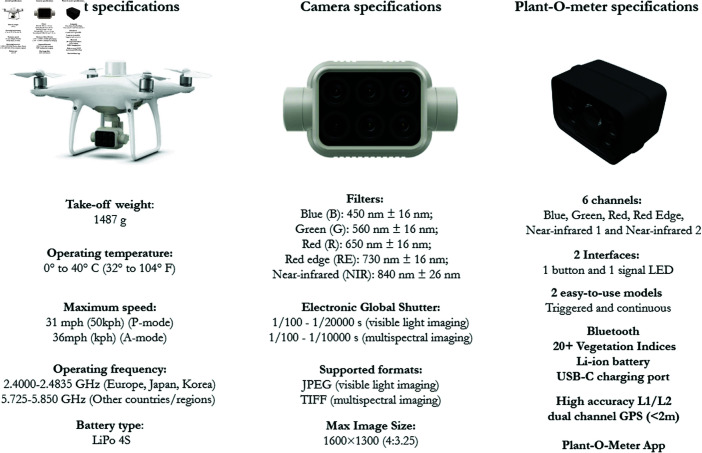
DJI Multispectral Aircraft, UAV camera and Plant-O-meter specifications.

A DJI P4 Multispectral quadcopter (Shenzhen, China) was used to capture nadir images of wheat cultivars under varying fertilization and seeding conditions. The main characteristics of the UAV platform and the camera are given in Fig 3. The UAV was flown at an altitude of 20 m, achieving a ground sampling distance (GSD) of 1.1 cm. Measurements were conducted between 11:00 and 14:00, under mostly sunny conditions, to minimize the impact of variations in solar radiation. A single battery cycle was sufficient to cover the entire trial area, and a total of 1,465 images were collected per sampling date. The DJI RTK2 mobile station provided real-time corrections, ensuring centimeter-level positional accuracy. UAV flights were approved, and the necessary permissions were issued by the Civil Aviation Authority [31] and the Ministry of Defense of the Republic of Serbia [32]. The collected multispectral images were processed using Pix4Dmapper (Switzerland), Version 4.8.2, with default settings, to generate high-resolution georeferenced orthomosaics across five spectral bands: blue, green, red, near-infrared (NIR), and red edge. The internal sun sensor of the Phantom drone was used for a relative radiometric calibration, ensuring consistency across flights. The Pix4Dmapper workflow included generating keypoints, matching them to create tie points, and constructing a point cloud. This point cloud was then used to develop the digital elevation model (DEM) and final orthomosaics. To assess crop condition and variability, a total of 19 vegetation indices (VIs) were calculated from the UAV-derived multispectral data. Zonal statistics (mean, median, max, and min values) were extracted for each plot to facilitate comparisons across different phenological stages. For accurate image registration across multiple time points, Global Mapper was used to manually add matching tie points (MTPs), as automatic alignment was not feasible due to significant variations in crop conditions throughout the growth cycle, which hindered consistent feature detection.

#### Plant-O-Meter

The Plant-O-Meter is a handheld, active multispectral sensor developed by the BioSense Institute and later commercialized by Bitgear (Belgrade, Serbia). It captures reflectance in six spectral bands (Blue at 455 nm, Green at 528 nm, Red at 657 nm, Red Edge at 740 nm, NIR1 at 810 nm, and NIR2 at 940 nm) and computes over 20 vegetation indices. The device is widely used in precision agriculture for detecting plant stress due to drought, heat, nutrient deficiencies, and pest pressure [33,34]. While limited in spatial coverage compared to aerial sensors, its high spatial resolution and flexible acquisition timing make it a valuable tool for localized assessments [35]. Measurements with the Plant-O-Meter were conducted between 11:00 and 14:00 to minimize variations in solar radiation. However, the device is equipped with an integrated light source, which helps minimize the influence of fluctuating ambient light conditions, ensuring consistent performance across measurements. For each genotype, spectral reflectance measurements were taken at four key growth stages: Booting - mid boot stage (BBCH43), Flag leaf completely emerged (BBCH49), Full flowering (BBCH65), and Medium milk (BBCH75) (see Fig 1). The measurement dates were determined based on the need to collect multispectral camera data at each stage of wheat development, including leaf and tiller development, stem elongation, heading and flowering, grain filling, and ripening. Additionally, at least three imaging sessions were scheduled before reseeding, which was made based on the observed field results, considering the crop performance and environmental conditions. The timing for Plant-O-Meter measurements was guided by literature findings, which highlight the flowering stage as the most effective for yield prediction using multispectral imaging. Studies indicate that features such as red reflectance and the NDRE vegetation index (VI) achieve high accuracy in yield prediction during this stage [36,37]. While the booting stage is less effective than the flowering stage, it still provides valuable data for yield estimation. In particular, the contrast texture feature during the booting stage demonstrated reasonable performance [38]. As the Milk stage (T8) is close to the Full flowering (T7) we made sure to cover it as well.

In-field reflectance measurements were conducted by holding the Plant-O-Meter sensor 70 cm horizontally above the crop canopy. Each wheat plot was scanned for approximately ten seconds, yielding over 30 spectral readings per plot. The mean value of these readings was calculated using the arithmetic mean, with outliers excluded based on a standard deviation threshold (values outside ±2 standard deviations from the mean were considered outliers and removed from the analysis).

### Vegetation indices

**Fig 4:**
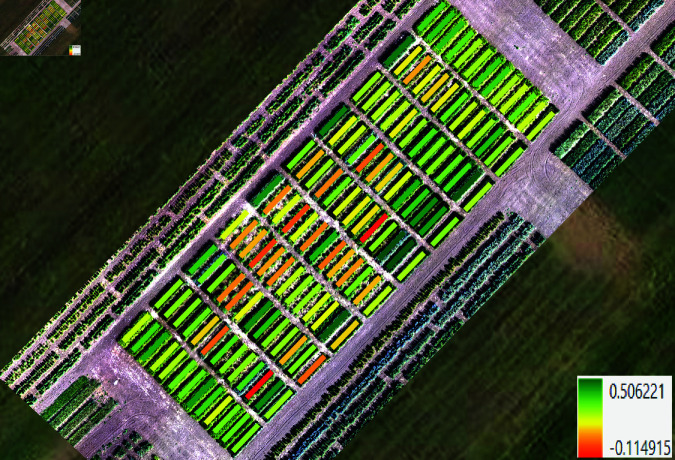
NDVI calculated per each plot.

The primary objective of computing vegetation indices was to analyze their behavior at different phenological stages and identify meaningful relationships. Fig 4 presents the NDVI per plot, calculated from the UAV multispectral data. A total of 34 vegetation indices were initially computed using the Plant-O-Meter. To ensure robust genotype selection at key growth stages, we focused on indices that showed minimal saturation at extreme values. Consequently, 19 indices met these criteria and were selected for further analysis, including a direct comparison with indices derived from UAV imagery. These indices included GARI, GCI, GLI, GOSAVI, GRDVI, GSAVI, IPVI, NDRE, NDVIb, NDVIg, NDVI, NDWI, NPCI, PNDVI, PSRI, RBNDVI, SAVI, VARI, and WDRVI (for full equations, see S3 Table.) To analyze spatial variability, index maps, zonal statistics, and time-series data were generated using a Python script in ArcGIS Pro [39–42] with the arcpy library [43]. This approach leveraged ArcGIS Pro’s geospatial analysis capabilities (such as zonal statistics, georeferencing, and alignment), allowing efficient and automated processing of spectral data.

### Genotype groups according to fertilization treatments and seeding cycles

The field trial design consisted of 130 subplots, each containing a specific genotype of the grass crop. Among these genotypes, 15 of them were resown: 40/I, 8/II mrk, 8/I-1, 1/I, 2/I, 3/I, 5/I, 6/Igr, Nirvana, Ostro, ZP Admiral, LP2-1-10, LP2-1-15, Emmer FON, and Populacija AU with altered fertilization and seeding cycles to observe their impact on yield. To facilitate a systematic analysis, subplots were categorized into four distinct groups based on specific fertilization and seeding cycles (see Schematic representation of the field trial design, S1 Figure). This approach allowed for a structured evaluation of how different fertilization regimes and seeding cycles influence crop performance. By comparing genotypes across these groups, the study aimed to identify optimal agronomic practices and determine the extent to which genotype-specific responses are affected by these treatments. The details of the fertilization and seeding cycles for each group are provided in Table 1.

**Table 1:** Genotype groups according to fertilization treatments and seeding cycles

Genotype groups according to fertilization treatments and seeding cycles		
No of group	Fertilization treatments	Seeding cycles
Group1	1	1
Group2	2	1
Group3	1	2
Group4	2	2

### In-field ground-truthing measurements

At full maturity, the grain yield (t/ha) and the plant height (cm) of each wheat genotype were estimated (See full list of genotypes, yields and plant heights in S4 Table). Plants from a $1\;m^{2}$ area of each genotype were harvested individually from the experimental plots to determine above-ground biomass (${g/m}^{2}$). Grain yield-related traits were also assessed, with final grain yield extrapolated to 14% moisture content. These measurements were conducted by researchers with domain knowledge from the Breeding Department, Maize Research Institute Zemun Polje, Belgrade.

## Results

### The impact of fertilization and seeding on crop yield

The optimal wheat development stages for yield prediction using multispectral imagery occur primarily during grain filling and flowering. Based on this, our analysis focuses on four key growth stages: full flowering (BBCH65), medium milk (BBCH75), early dough (BBCH83), and full ripeness (BBCH89). Considering the previously defined genotype groupings, we evaluated the effects of different fertilization levels and seeding cycles on crop yield. Our analysis revealed that, with some exceptions, nitrogen application showed weak correlations with biophysical parameters at all growth stages. Table 2 presents the effect of fertilization and seeding on yield. The results indicate that genotypes subjected to high fertilization treatments achieved the highest yields, followed by those under low fertilization treatments. Additionally, reseeded genotypes consistently outperformed those that were not reseeded.

**Table 2:** The best-performing genotypes according to yield

Genotype	High Fertilizer	Low Fertilizer	Reseeding	No Reseeding
BLT 34-15	5730 (G1)	4028 (G2)	5061 (G3)	4111 (G4)
BLR 8-15	5356 (G1)	4780 (G2)	4886 (G3)	4500 (G4)
NS Dur	4238 (G1)	4210 (G2)	4300 (G3)	3983 (G4)
Agaton	4028 (G1)	3755 (G2)	4188 (G3)	3340 (G4)
Bambi	3760 (G1)	3118 (G2)	4200 (G3)	3480 (G4)
Odisej	3530 (G1)	4437 (G2)	4335 (G3)	4120 (G4)
Cosmostar	2720 (G1)	3900 (G2)	3869 (G3)	3400 (G4)

Y1–Y4 represent yield ranking from highest (Y1) to lowest (Y4).

**Fig 5:**
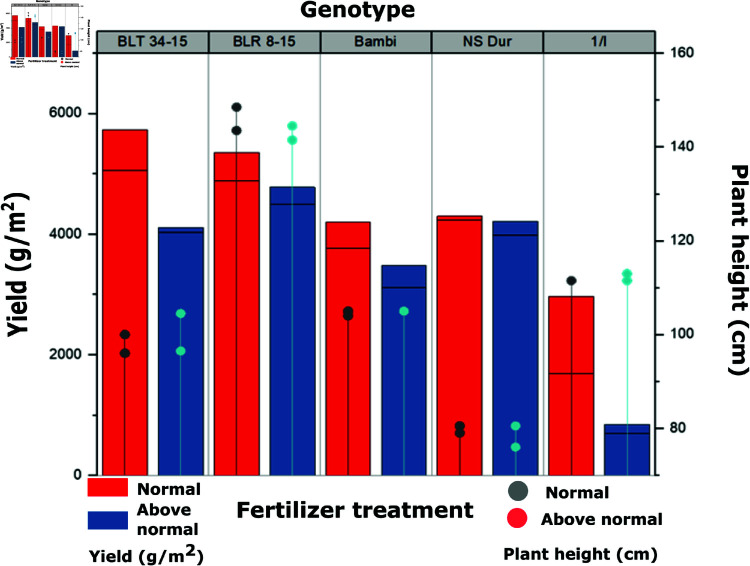
The analysis of yield and plant height across different genotypes and treatment types.

Moreover, the analysis of yield and plant height across different genotypes and treatment types reveals a consistent trend. The normal fertilization treatment produced the highest yield and maximum plant height across all varieties. In contrast, the above-normal treatment, which involves a double application of fertilizer, resulted in an increase in plant height but a noticeable decline in yield across all varieties and genotypes. This suggests that while additional fertilizer application enhances vegetative growth (biomass accumulation), it does not translate into higher grain yield. Excessive nutrient availability likely shifts the plant’s energy allocation toward vegetative growth rather than reproductive development, leading to reduced grain formation. This trend is illustrated in Fig 5, where varieties (shown in clustered bars) under the normal treatment consistently outperform their counterparts under the above-normal treatment in terms of yield.

Furthermore, plant height variations indicate that while additional fertilizer contributes to taller plants, this does not necessarily correlate with improved yield efficiency. This suggests a possible nutrient imbalance, where excess fertilization leads to excessive stem elongation, potentially making plants more susceptible to lodging and reducing their overall productivity. These results highlight the value of integrating remote sensing tools to optimize nitrogen management, improving yield results and minimizing environmental risks.

### The identification of high- and low-performing cereal genotypes across various growth stages

**Fig 6:**
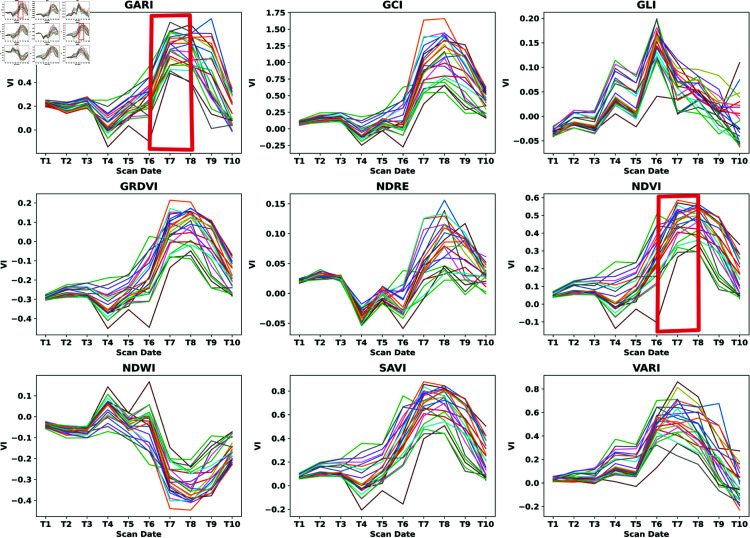
Spectral reflectance measurements across nine vegetation indices (VIs) for all genotypes, highlighting the T6 and T7 as optimal stages for measurement.

Vegetation indices revealed significant variations in index values at key phenological stages. Specifically, noticeable shifts were observed when the flag leaf had fully emerged (T6) and at the full flowering stage (T7) (see Fig 6). These changes highlight the importance of these growth stages for assessing plant health and performance using spectral data. Comparing UAV-derived vegetation indices with handheld sensor measurements allowed for the identification of high- and low-performing genotypes (see Fig 1)). The results demonstrate that spectral indices obtained from UAV imagery were highly effective in distinguishing performance differences across genotypes. Furthermore, correlations between UAV and Plant-O-Meter data confirmed the reliability of remote sensing techniques in evaluating crop conditions at critical growth stages (see Fig 6).

**Fig 7:**
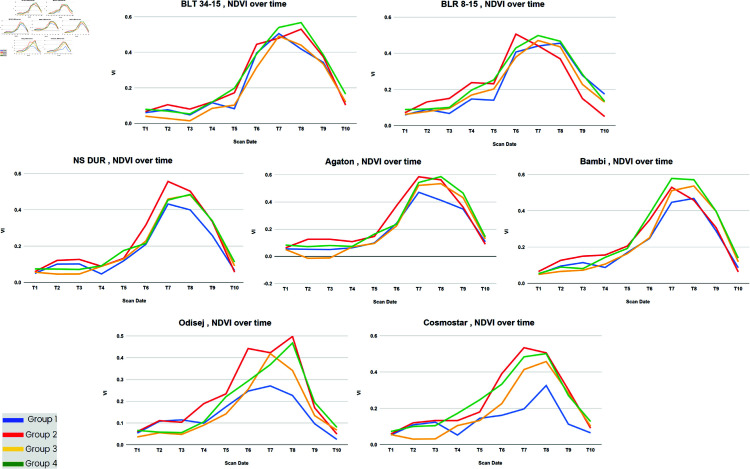
The comparison of the best-performed genotypes from all four groups using NDVI.

These stages were further analyzed to identify genotypes with high performance in terms of yield. The top genotypes with the highest yield from each group were compared with the top genotypes exhibiting the highest vegetation values at the booting and spike emergence stages. Notably, it was observed that several genotypes with the highest vegetation index at these stages also yielded the highest crop yield across all plots. The best-performing genotypes according to yield vs VI were shown in Fig 7 using NDVI. The findings from the study, suggest that the identified stage where the flag leaf has completely emerged (T6) and full flowering stage (T7) (see Fig 6) could be the most optimal stages to conduct the spectral reflectance measurement for early prediction of grain yield. This means they can serve as indicators of the future yield potential of large germplasm and genotypes. These results are similar to those obtained by Liu et al, 2019 [44], who also observed the significance of spectral reflectance measurement in the heading stage of wheat in predicting grain yield. In Ljubicic et al, [45] different priming conditions of wheat seed revealed that the most indicative stage for SR measurements was the full flowering stage of wheat for stem height. In contrast, for the trait spike length of wheat, the greatest positive association was found at the medium milk-growing stage. In the tables below (Table 3,Table 4,Table 5 and Table 6 are presented the highest-performing yield genotypes in T6 and T7 growth stages. The analysis provides valuable insights into genotype consistency, the effects of different fertilization treatments, and the impact of reseeding.

**Table 3:** Highest performing yield genotypes in T6 and T7 growth stages: The first seeding cycle and oen fertilization treatment—Group 1

Genotypes	Top10 of T6	Top10 of T7
BLT 34-15	√	√
Sofru	√	√
BLR 8-15	√	√
P4		
P5		
P3		
P2		
Bordeaux	√	√
Ilico		
P1		

**Table 4:** Highest performing yield genotypes in T6 and T7 growth stages: The first seeding cycle with two fertilization treatments—Group 2

Genotypes	Top10 of T6	Top10 of T7
BLR 8-15	√	
Odisej	√	
NS Dur	√	√
BLT 34-15	√	√
Cosmostar	√	√
Agaton	√	√
ZP Admiral		
Bambi	√	√
LP2-1-15		
5/1		

**Table 5:** Highest performing yield genotypes in T6 and T7 growth stages: The reeseding with one fertilization treatment—Group 3

Genotypes	Top10 of T6	Top10 of T7
RGA 5	√	√
BLT 34-15	√	
BLR 8-15	√	
Bordeaux	√	√
RGA 4	√	√
RGA 2		
Odisej		
RGA 4	√	√
RGA 1		

**Table 6:** Highest performing yield genotypes in T6 and T7 growth stages: The reseeding with two fertilization treatments—Group 4

Genotypes	Top10 of T6	Top10 of T7
BLR 8-15	√	√
Odisej	√	√
BLT 34-15	√	√
Bambi	√	√
Cosmostar	√	√
Agaton	√	√
ZP Admiral		
8/1-1	√	√
LP2-1-15		

Genotypes such as BLT 34-15, Sofru, BLR 8-15, and Bordeaux consistently appeared in both T6 and T7, indicating their robustness across both growth stages and their ability to perform well under a single fertilization treatment. The lack of performance for genotypes like P4, P5, and Ilico in both stages suggests that they might require alternative conditions (e.g., additional fertilization or different seeding strategies) to perform optimally. Genotypes in Group 1 that consistently performed well in both T6 and T7, such as BLT 34-15 and Sofru, could be prime candidates for further studies to explore their resilience to fertilization treatments.

This group highlights the importance of fertilization treatment in boosting yields, with genotypes like NS Dur and Cosmostar standing out as adaptable and resilient under dual treatments. However, further testing is required for genotypes like ZP Admiral to better understand the factors influencing their growth.

In Group 3, RGA 5, BLT 34-15, and Bordeaux showed consistent performance across T6 and T7, indicating their strong adaptation to reseeding practices combined with a single fertilization treatment. Meanwhile, RGA 2 and 4 performed well in T6, but RGA 2 did not show similar results in T7. This may suggest that reseeding provides benefits in the early stages, but its effectiveness might diminish over time. On the other hand, genotypes like Odisej and RGA 1 showed limited or no performance, pointing towards possible sensitivities to reseeding practices or their specific fertilization treatment needs and may require additional adjustments to improve consistency across growth stages.

From the last Group 4, BLR 8-15, Odisej, Bambi, and Cosmostar demonstrated strong performance in both T6 and T7, indicating that reseeding with two fertilization treatments is highly effective for these genotypes. Agaton also showed strong consistency across both growth stages, further supporting the idea that dual fertilization treatments enhance genotype performance, particularly under reseeding conditions. ZP Admiral and LP2-1-15 did not appear in the top-performing genotypes for T7, suggesting that the combination of reseeding and two fertilization treatments does not necessarily improve yields for all genotypes. The overall findings suggest that genotypes in Groups 3 and 4, which involve reseeding, generally showed better consistency across growth stages (T6 and T7) than those in Groups 1 and 2, where reseeding was not performed. This suggests that reseeding may provide an advantage in optimizing yield performance under specific fertilization treatments. According to the presence of two fertilization treatments (Groups 2 and 4) generally resulted in higher and more consistent yields, with Cosmostar, Agaton, and NS Dur emerging as robust performers under dual fertilization. However, some genotypes like P4 and P5 in Group 1 performed poorly, indicating that their optimal growth conditions might not be fully met by either of the fertilization treatments used in the study. Some genotypes, particularly BLT 34-15, Sofru, and Bordeaux, stood out across multiple growth stages and treatments. This highlights the potential of these genotypes for further research and practical application in agricultural practices, especially under varying fertilization and seeding conditions. This analysis emphasizes the complex interaction between fertilization treatments, seeding practices, and genotype performance. Genotypes like BLT 34-15 and Sofru demonstrate strong adaptability across both growth stages and different treatment conditions, making them ideal candidates for future studies. Additionally, reseeding combined with dual fertilization has shown promising results, particularly for BLR 8-15 and Odisej. Understanding the factors contributing to the performance of these genotypes could lead to more efficient agricultural practices and improved crop yields. The overall findings suggest that the identified vegetation indices at the booting and spike emergence stages can serve as indicators of the future yield potential of different genotypes. By leveraging UAV-based vegetation indices, it is possible to assess and select genotypes with favorable characteristics early in the crop cycle, enabling efficient resource allocation and crop management strategies. This information can be further used in breeding programs for selecting high-yielding genotypes under similar agroecological conditions. The proposed approach holds promise for enhancing breeding programs and optimizing agricultural practices by identifying high-performing genotypes at critical stages of crop development based on their vegetation index values. Furthermore, the identification of significant VIs in the boot stage could be beneficial for making fungicide applications for foliar disease management, particularly late-season diseases [48].

### The comparison between Plant-O-Meter and UAV data

The research paper incorporated the use of a handheld proximal sensor, Plant-O-Meter, for data acquisition to monitor vegetation indices in each plot. Data from the Plant-O-Meter was collected at four stages of the crop cycle: Booting - mid boot stage (T5), flag leaf emergence (T6), full flowering (T7), and medium milk (T8). The acquired Plant-O-meter data was compared with the vegetation indices generated using UAV-based data. The vegetation indices (VIs) that exhibited the strongest Pearson correlation between Plant-O-Meter and UAV measurements were GRDVI, NDVI, and SAVI, with p-values below 0.05, indicating statistical significance. Among the selected genotypes representing different performance levels—RGA 3 (low), BLT 34-15 (mid), and BLR 8-15 (high)—VIs consistently demonstrated a very strong positive correlation. The obtained results (See Table 7) showed the average Pearson correlation values of GRDVI, NDVI, and SAVI: 0.957, 0.954 and 0.944.

**Table 7:** Pearson correlation of VIs across selected genotypes

Genotype	GRDVI	NDVI	SAVI
RGA 3 (Low)	0.926	0.896	0.904
BLT 34-15 (Mid)	0.954	0.975	0.988
BLR 8-15 (High)	0.992	0.993	0.941
Average Correlation	0.957	0.954	0.944

Despite the partial lack of calibration of the Plant-O-Meter sensors to a common object reflectance value, it was observed that the values obtained from the Plant-O-Meter were generally higher. Furthermore, the trend of value changes in both the UAV-based and Plant-O-Meter data exhibited a similar pattern. Even though, the study focused on identifying the top 20 genotypes with high vegetation indices values using UAV-based data and comparing them to the genotypes with high vegetation indices detected by the Plant-O-Meter. Notably, there was a match of 16 genotypes out of the top 20, indicating an 80% agreement between the two data acquisition methods. This finding suggests that both the UAV and the Plant-O-Meter can be used for similar tasks, although they have distinct tradeoffs in terms of data acquisition and processing.

## Discussion

**Fig 8:**
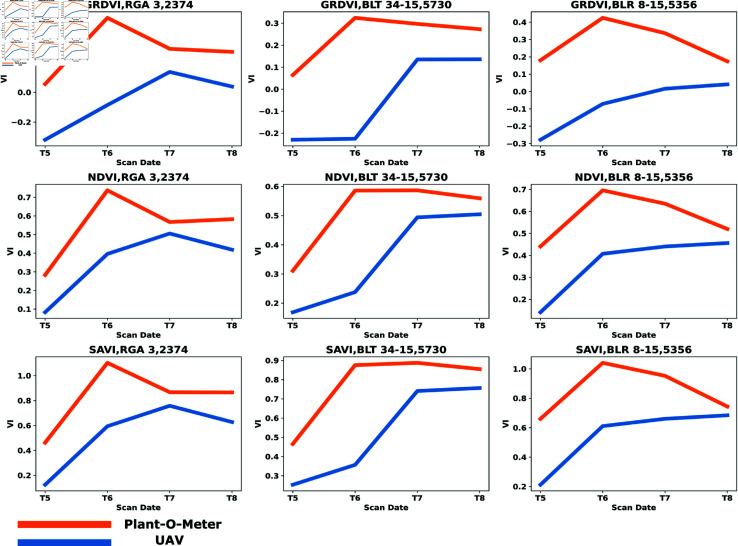
Comparison between Plant-O-Meter and UAV data for three genotypes: RGA3, BLT 34-15, and BLR 8-15, according to three vegetation indices: GRDVI, NDVI, and SAVI.

The primary goal of this research was to investigate the effects of varying fertilization doses and seeding application rates on the yield of 41 different grass crop genotypes, alongside comparing the performance of UAV and proximal sensors for crop monitoring. By experimenting with 130 plots and applying different fertilization cycles and seeding doses, we aimed to identify optimal strategies for maximizing crop yield. Our findings revealed significant genotype-specific responses to fertilization and seeding rates, underscoring the need for tailored agricultural practices for each genotype. These results highlight the importance of fine-tuning fertilization and seeding strategies in real-world agricultural settings. In agreement with the study [46], the variability in nitrogen use efficiency across different genotypes under varying fertilization treatments mirrors our observation that genotype plays a key role in yield response to fertilization. Similarly, as in [47], our results showed that the impact of fertilization and seeding cycles on crop yield was genotype-dependent. Specifically, some genotypes responded positively to increased fertilization cycles, while others demonstrated a decrease in yield under the same conditions. This suggests that certain genotypes may be sensitive to excessive fertilization, which can negatively impact productivity. These findings support the notion that optimizing fertilization strategies based on genotype-specific needs is crucial, especially in regions with varying soil conditions. For genotypes that showed a decrease in yield with higher fertilization doses, lower nitrogen levels may be more suitable, while higher fertilization rates could benefit genotypes that responded positively to increased nutrient availability. This genotype-specific approach is crucial for maximizing yield and minimizing environmental impacts associated with overfertilization. Similarly, seeding application rates also exhibited genotype-specific responses, with some genotypes benefiting from higher seeding doses, while others showed reduced yields due to seedling overcrowding and competition for resources. These results suggest that precise seeding rates should be determined for each genotype to optimize yield potential. This is particularly relevant in commercial wheat production, where farmers can improve productivity by adjusting both fertilization and seeding strategies based on genotype characteristics. The strong Pearson correlations between vegetation indices (GRDVI, NDVI, and SAVI) obtained from both Plant-O-Meter and UAV measurements further support the reliability of these methods for monitoring crop performance. The identification of significant VIs during the booting stage holds practical value for early yield potential estimation and decision-making in crop management, especially for managing disease risks and determining optimal fertilization strategies. These results are in line with previous studies that have highlighted the role of vegetation indices in early-stage yield prediction and disease management [48]. In this study, GRDVI, NDVI, and SAVI reached the highest Pearson correlation between Plant-O-Meter and UAV measurements. Among the selected genotypes representing different performance levels, GRDVI and NDVI reached the highest correlation value for BLR 8-15: 0.992 and 0.993, while SAVI reached the highest value for BLT 34-15: 0.988. This suggests that VIs values obtained from both methods are highly comparable, reinforcing their reliability for monitoring crop performance. Additionally, the identification of significant VIs during the booting stage is valuable for early yield potential estimation and decision-making in disease management, particularly for fungicide applications targeting late-season foliar diseases [48]. In Fig 8, blue represents VI values obtained by UAV, while orange represents those measured by the Plant-O-Meter, visually illustrating the level of agreement between the two methods.

In terms of data acquisition, UAV-based methods offer high-resolution spatial data but come with certain challenges, including the need for skilled operators and large volumes of data that require advanced processing techniques. UAV surveys are subject to factors such as weather conditions and unforeseen obstacles, which can complicate the data collection process. However, UAVs provide high-resolution maps that are essential for detailed spatial analysis and yield prediction. Conversely, Plant-O-Meter-based data acquisition is more straightforward and less resource-intensive. While it provides less spatial detail, it offers direct, plot-specific measurements that are valuable for vegetation index calculation. The choice between UAV and Plant-O-Meter depends on factors such as the need for spatial resolution, the availability of skilled personnel, and the volume of data required for analysis. Combining both methods may offer complementary strengths for more comprehensive crop monitoring. While the primary focus of this study was on the effects of fertilization and seeding strategies, we also observed that ancient cereal species, particularly when cultivated under less favorable conditions, demonstrated notable climatic adaptation and resilience to both abiotic and biotic stressors. These research findings confirm the potential of certain ancient cereal species cultivated under less favorable conditions to offer a sustainable alternative, demonstrate enhanced climatic adaptation, increase resistance to abiotic and biotic stressors, and contribute to greater biodiversity. The study underscores the valuable advantages ancient cereal crops hold over their contemporary counterparts, suggesting their significance in promoting agricultural resilience and sustainability in the face of evolving environmental challenges.

### Study limitations and future directions

This study provides valuable insights into genotype-specific responses to fertilization cycles and seeding application rates. However, several limitations should be acknowledged. The analysis was conducted on 41 cereal crop genotypes, which, while informative, represents only a subset of the genetic diversity present in cereal crops. Expanding the study to include more genotypes across multiple locations and agroecological conditions would enhance the unalterability and robustness of the findings. Environmental factors such as soil quality, weather variability, and pest pressures may have influenced the results and should be carefully controlled in future research. Additionally, integrating more advanced data analysis techniques, such as machine learning algorithms, could improve predictive modeling and optimize genotype-specific recommendations. In terms of data collection, UAV-based monitoring requires skilled operators, large data storage, and complex image processing workflows. The development of more automated and user-friendly data processing pipelines could reduce these challenges. Moreover, image registration remains a significant hurdle due to changing crop conditions over time. The incorporation of physical markers throughout the crop cycle could improve UAV image alignment and facilitate more consistent data analysis. Future studies should explore these approaches to enhance the efficiency and accessibility of UAV-based phenotyping.

## Conclusion

This study demonstrates the genotype-specific responses of 41 different cereal crop genotypes to variations in fertilization cycles and seeding application rates. Our findings highlight the necessity of tailored fertilization and seeding strategies to optimize crop yield while balancing nutrient availability and seed density. Implementing such genotype-specific approaches in wheat cultivation can contribute to sustainable agriculture by increasing productivity and minimizing resource waste. Additionally, this research underscores the potential of UAV-based data acquisition and the Plant-O-Meter for vegetation index monitoring. The strong correlation between indices obtained from both methods suggests their reliability for detecting high-performing genotypes. While UAVs offer high-resolution spatial analysis and frequent monitoring, Plant-O-Meter-based measurements provide a straightforward, less data-intensive alternative. A combined approach could further enhance plant phenotyping and precision agriculture strategies. Furthermore, our findings indicate that certain ancient cereal species cultivated under less favorable conditions exhibit enhanced climatic adaptation and resistance to abiotic and biotic stressors. This highlights their potential role in promoting agricultural resilience and sustainability in response to climate change. Future research should focus on expanding the study to include additional locations, genotypes, and fertilization regimes. Incorporating machine learning models and automation in data analysis could further refine predictive yield estimation. By adopting genotype-specific agronomic strategies and leveraging advanced monitoring technologies, researchers and farmers can contribute to more efficient and sustainable agricultural practices.

## Supporting information

S1 Table Measurement dates and data sources.
(MS Word)

S2 Table List of genotypes, species, and their scientific names.
(MS Word)

S3 Table 19 selected Vegetation Indices (VIs).
(MS Word)

S4 Table The table presents yield and plant height for different genotypes.
(MS Word)

S1 Fig Trial designs: fertilization and reseeding.
(TIFF)
