# Supplementary material for: Integrating UAV multispectral imaging and proximal sensing for high-precision cereal crop monitoring
Source: PLoS One. 2025 May 22;20(5):e0322712. doi: 10.1371/journal.pone.0322712 (PMC12097617; doi:10.1371/journal.pone.0322712)
Supplement: S1 Table — Measurement dates and data sources. (MS Word) [file pone.0322712.s001.docx]

Supporting information 519

S1 Table. Measurement Dates and Data Sources. 520

| **Notation** | **Date** | **UAV** | **Plant-O-Meter** |
| --- | --- | --- | --- |
| T1 | 17.11.2021. | X |  |
| T2 | 01.12.2021. | X |  |
| T3 | 24.12.2021. | X |  |
| T4 | 24.02.2022. | X |  |
| T5 | 18.03.2022. | X | X |
| T6 | 11.04.2022. | X | X |
| T7 | 16.05.2022. | X | X |
| T8 | 27.05.2022. | X | X |
| T9 | 07.06.2022. | X |  |
| T10 | 20.06.2022. | X |  |
