# Supplementary material for: Integrating UAV multispectral imaging and proximal sensing for high-precision cereal crop monitoring
Source: PLoS One. 2025 May 22;20(5):e0322712. doi: 10.1371/journal.pone.0322712 (PMC12097617; doi:10.1371/journal.pone.0322712)
Supplement: S2 Table — List of genotypes, species, and their scientific names. (MS Word) [file pone.0322712.s002.docx]

| **Genotype** | | **Species** | **Scientific name** |
| --- | --- | --- | --- |
| Brodeaux | 1 | Barley | Hordeum vulgare |
| NS Dur | 2 | Durum wheat | Triticum durum |
| Agaton | 3 | Durum wheat | Triticum durum |
| Cosmostar | 4 | Durum wheat | Triticum durum |
| Sofru | 5 | Wheat | Triticum aestivum |
| Bambi | 6 | Wheat - compactum | Triticum compactum |
| Ilico | 7 | Wheat | Triticum aestivum |
| BLT 34-14 | 8 | Triticale | Triticale |
| BLR 8-15 | 9 | Rye | Secale cereale |
| Odisej | 10 | Triticale | Triticale |
| Zp Admiral | 11 | Triticale | Triticale |
| P1 | 12 | Wheat | Triticum aestivum |
| P2 | 13 | Wheat | Triticum aestivum |
| P3 | 14 | Wheat | Triticum aestivum |
| P4 | 15 | Wheat | Triticum aestivum |
| P5 | 16 | Wheat | Triticum aestivum |
| RGA 1 | 17 | Barley | Hordeum vulgare |
| RGA 2 | 18 | Barley | Hordeum vulgare |
| RGA 3 | 19 | Barley | Hordeum vulgare |
| RGA 4 | 20 | Barley | Hordeum vulgare |
| RGA 5 | 21 | Barley | Hordeum vulgare |
| 8/I-1 | 22 | Spelt | Triticum spelta |
| LP 2-1-15 | 23 | Emmer (dvozrna wheat) | Triticum dicoccon |
| 40/I | 24 | Spelt | Triticum spelta |
| 8/II | 25 | Spelt | Triticum spelta |
| 1/I | 26 | Spelt | Triticum spelta |
| 2/I | 27 | Spelt | Triticum spelta |
| 3/I | 28 | Spelt | Triticum spelta |
| 5/I | 29 | Spelt | Triticum spelta |
| 6/Igr | 30 | Spelt | Triticum spelta |
| LP2-1-1 | 31 | Einkorn | Triticum monococcum |
| LP2-1-10 | 32 | Emmer (dvozrna wheat) | Triticum dicoccon |
| Emmer Fon | 33 | Emmer (dvozrna wheat) | Triticum dicoccon |
| Nirvana | 34 | Spelt | Triticum spelta |
| Isocel | 35 | Barley | Hordeum vulgare |
| SBE | 36 | Wheat | Triticum aestivum |
| W1 | 37 | Wheat | Triticum aestivum |
| Waxy 1 | 38 | Wheat | Triticum aestivum |
| 8/II mrk | 39 | Spelt | Triticum spelta |
| Tr. sphaerococcum | 40 | Shot wheat | Triticum sphaerococcum |
| Ostro | 41 | Spelt | Triticum spelta |
