# Supplementary material for: Integrating UAV multispectral imaging and proximal sensing for high-precision cereal crop monitoring
Source: PLoS One. 2025 May 22;20(5):e0322712. doi: 10.1371/journal.pone.0322712 (PMC12097617; doi:10.1371/journal.pone.0322712)
Supplement: S3 Table — 19 selected Vegetation Indices (VIs). (MS Word) [file pone.0322712.s003.docx]

| **Name** | **Equation** |
| --- | --- |
| Green Atmospherically Resistant Index (GARI) | ((NIR *−* Red) *−* (Green *−* Red))*/*((NIR *−* Red) + (Green *−* Red)) |
| Normalized Difference Vegetation Index (NDVI) | (NIR *−* Red)*/*(NIR + Red) |
| Green Chlorophyll Index (GCI) | Green*/*Red |
| Normalized Difference Water Index (NDWI) | (Green *−* NIR)*/*(Green + NIR) |
| Green Leaf Index (GLI) | 2 *×* Green *−* Red *−* Blue |
| Normalized Pigment Chlorophyll Index (NPCI) | (Green *−* Red)*/*(Green + Red) |
| Green Optimized Soil Adjusted Vegetation Index (GOSAVI) | (NIR *−* (2 *×* Red) + 1)*/*(NIR + (2 *×* Red) + 1) |
| Perpendicular Vegetation Index (PNDVI) | (NIR *−* Red)*/*(NIR + Red) |
| Plant Senescence Reflectance Index (PSRI) | (Red *−* NIR)*/*(Red + NIR) |
| Red-Blue Normalized Difference Vegetation Index (RBNDVI) | (Red *−* Blue)*/*(Red + Blue) |
| Soil Adjusted Vegetation Index (SAVI) | ((1 + *L*) *×* (NIR *−* Red))*/*(NIR + Red + *L*) |
| Visible Atmospherically Resistant Index (VARI) | (Green *−* Red)*/*(Green + Red *−* Blue) |
| Wide Dynamic Range Vegetation Index (WDRVI) | (0*.*1 *×* NIR *−* Red)*/*(0*.*1 *×* NIR + Red) |
| Infrared Percentage Vegetation Index (IPVI) | (NIR *−* Red)*/*NIR |
| Normalized Difference Red Edge (NDRE) | (NIR *−* Red Edge)*/*(NIR + Red Edge) |
| Normalized Difference Vegetation Index Blue (NDVIb) | (NIR *−* Blue)*/*(NIR + Blue) |
| Normalized Difference Vegetation Index Green (NDVIg) | (NIR *−* Green)*/*(NIR + Green) |
| Green-Red Difference Vegetation Index (GRDVI) | (Green *−* Red)*/*(Green + Red) |
| Green Soil Adjusted Vegetation Index (GSAVI) | (NIR *−* Green)*/*(NIR + Green + 0*.*5) |

**Notes:** NIR = Near-Infrared, L = Soil brightness correction factor, Red Edge = specific spectral band. Indices are used for vegetation monitoring.
