# Supplementary material for: Integrating UAV multispectral imaging and proximal sensing for high-precision cereal crop monitoring
Source: PLoS One. 2025 May 22;20(5):e0322712. doi: 10.1371/journal.pone.0322712 (PMC12097617; doi:10.1371/journal.pone.0322712)
Supplement: S4 Table — The table presents yield and plant height for different genotypes. (MS Word) [file pone.0322712.s004.docx]

S4 Table. The table presents yield and plant height for different 524

**genotypes.** 525

| **Genotype** | **Yield (g/m^2^)** | **Plant height (cm)** |
| --- | --- | --- |
| P1 | 4372 | 75 |
| P2 | 4559 | 75 |
| P3 | 4600 | 61 |
| P4 | 5065 | 73.5 |
| P5 | 4656 | 64 |
| Waxy 1 | 2605 | 71.5 |
| W1 | 2589 | 64 |
| SBE | 1322 | 61 |
| Ilico | 4500 | 73.5 |
| Sofru | 5387 | 65 |
| Bordeaux | 4877 | 74 |
| BLT 34-15 | 4111 | 96.5 |
| Agaton | 3340 | 85 |
| BLR 8-15 | 4500 | 141.5 |
| Populacija AU | 388 | 116 |
| BLT 34-15 | 4028 | 104.5 |
| Agaton | 3755 | 88 |
| BLR 8-15 | 4780 | 144.5 |
| Populacija AU | 1240 | 116 |
| Isocell | 2035 | 66 |
| RGA 1 | 2815 | 63.5 |
| 40/I | 980 | 128 |
| 3/I | 1167 | 124 |
| Cosmostar | 3900 | 82.5 |
| Odisej | 4437 | 105.5 |
| 2/I | 880 | 119 |
| Nirvana | 508 | 110.5 |
| ZP Admiral | 2304 | 94 |
| LP2-1-15 | 1090 | 118 |
| RGA 1 | 3965 | 77.5 |
| RGA 2 | 3220 | 71.5 |
| Odisej | 4120 | 96 |
| NS Dur | 3983 | 76 |
| 3/I | 798 | 110 |
| 8/II mrk | 434 | 109.5 |
| LP2-1-1 | 938 | 112 |
| NS Dur | 4210 | 80.5 |
| 5/I | 1540 | 121.5 |
