# Supplementary figures and images for: Integrating UAV multispectral imaging and proximal sensing for high-precision cereal crop monitoring
Source: PLoS One. 2025 May 22;20(5):e0322712. doi: 10.1371/journal.pone.0322712 (PMC12097617; doi:10.1371/journal.pone.0322712)

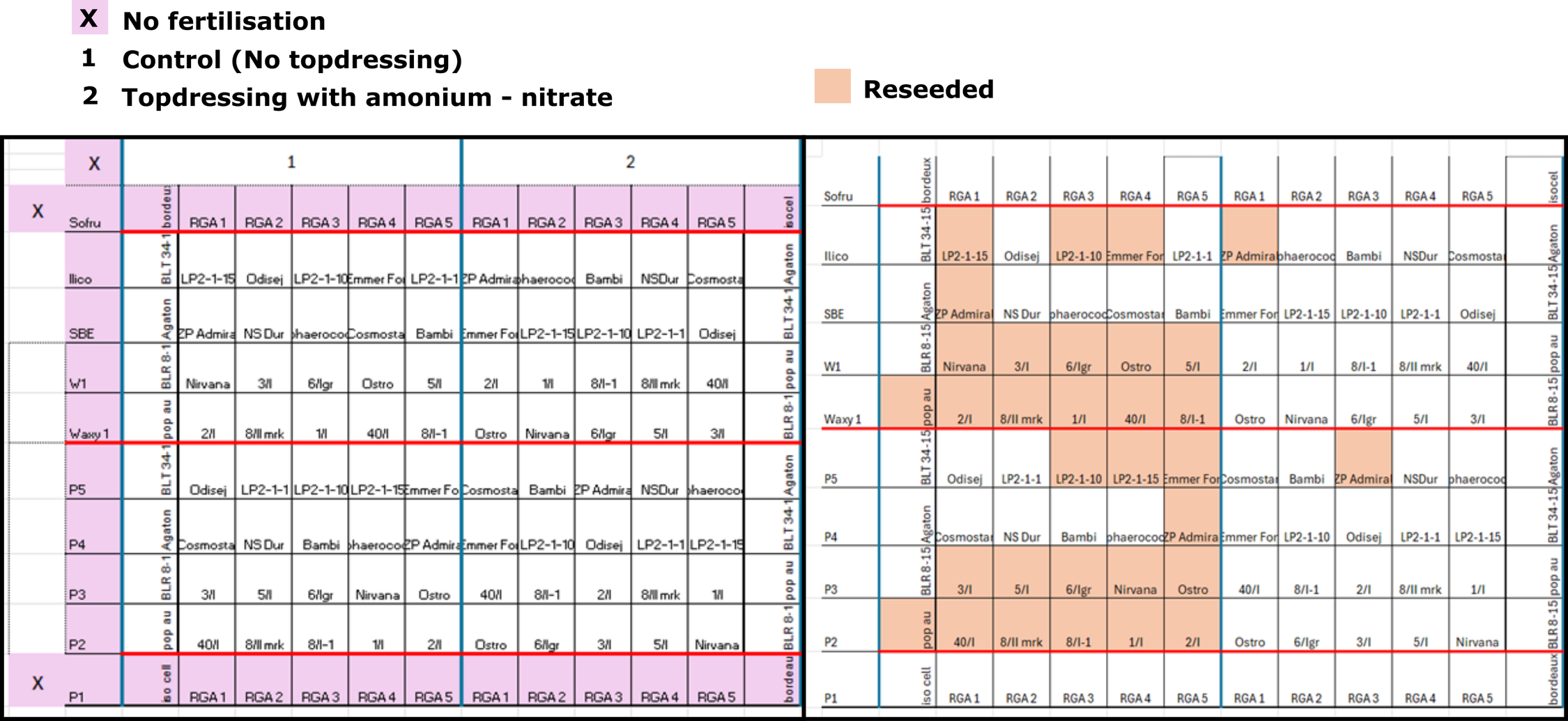

Supplement: S1 Fig — Trial designs: fertilization and reseeding. (TIFF) [file pone.0322712.s005.tif]
